# Comparative gene expression profiling in two congenic mouse strains following *Bordetella pertussis *infection

**DOI:** 10.1186/1471-2180-7-88

**Published:** 2007-10-12

**Authors:** Sander Banus, Rob J Vandebriel, Jeroen LA Pennings, Eric R Gremmer, Piet W Wester, Henk J van Kranen, Timo M Breit, Peter Demant, Frits R Mooi, Barbara Hoebee, Tjeerd G Kimman

**Affiliations:** 1 Laboratory for Infectious Diseases and Screening, National Institute of Public Health and the Environment (RIVM), PO Box 1, 3720 BA Bilthoven, The Netherlands; 2 Laboratory of Toxicology, Pathology, and Genetics, National Institute of Public Health and the Environment (RIVM), PO Box 1, 3720 BA Bilthoven, The Netherlands; 3 Microarray Department (MAD), Swammerdam Institute for Life Sciences, Faculty of Science, University of Amsterdam, The Netherlands; 4 Department of Molecular and Cellular Biology, Roswell Park Cancer Institute, Buffalo, New York 14263, USA

## Abstract

**Background:**

Susceptibility to *Bordetella pertussis *infection varies widely. These differences can partly be explained by genetic host factors. HcB-28 mice are more resistant to *B. pertussis *infection than C3H mice, which could partially be ascribed to the *B*. *pertussis susceptibility locus-1 *(*Bps1*) on chromosome 12. The presence of C57BL/10 genome on this locus instead of C3H genome resulted in a decreased number of bacteria in the lung. To further elucidate the role of host genetic factors, in particular in the *Bps1 *locus, in *B. pertussis *infection, and to identify candidate genes within in this region, we compared expression profiles in the lungs of the C3H and HcB-28 mouse strains following *B. pertussis *inoculation. Twelve and a half percent of the genomes of these mice are from a different genetic background.

**Results:**

Upon *B. pertussis *inoculation 2,353 genes were differentially expressed in the lungs of both mouse strains. Two hundred and six genes were differentially expressed between the two mouse strains, but, remarkably, none of these were up- or down-regulated upon *B. pertussis *infection. Of these 206 genes, 17 were located in the *Bps1 *region. Eight of these genes, which showed a strong difference in gene expression between the two mouse strains, map to the immunoglobulin heavy chain complex (*Igh*).

**Conclusion:**

Gene expression changes upon *B. pertussis *infection are highly identical between the two mouse strains despite the differences in the course of *B. pertussis *infection. Because the genes that were differentially regulated between the mouse strains only showed differences in expression before infection, it appears likely that such intrinsic differences in gene regulation are involved in determining differences in susceptibility to *B. pertussis *infection. Alternatively, such genetic differences in susceptibility may be explained by genes that are not differentially regulated between these two mouse strains. Genes in the *Igh *complex, among which *Igh-1a/b*, are likely candidates to explain differences in susceptibility to *B. pertussis*. Thus, by microarray analysis we significantly reduced the number of candidate susceptibility genes within the *Bps1 *locus. Further work should establish the role of the *Igh *complex in *B. pertussis *infection.

## Background

*Bordetella pertussis *is a gram-negative bacterium that can cause the respiratory disease known as pertussis or whooping cough in humans. Susceptibility to this disease and its course vary widely between individuals [1]. We have previously shown that genetically divergent mouse strains differ in their response to *B. pertussis *infection, underlining that infection is influenced by host genetic factors[2,3]. In addition, a role of several host genetic loci in the course of *B. pertussis *infection has been indicated, such as the toll-like receptor 4 (Tlr4) gene [2-5], the interferon gamma receptor gene [6], and three novel loci, *B. pertussis susceptibility locus 1, 2, and 3 (Bps1, 2, and 3) *[2] that showed linkage with the severity of infection.

**Figure 1:**
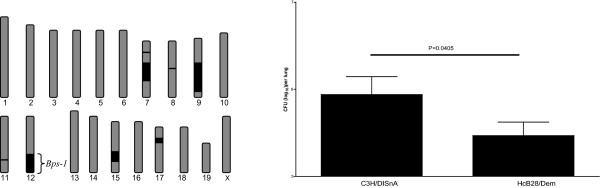
**Differences between C3H/DISnA and HcB-28/Dem mice**. a) Illustration of the distribution pattern of B10 genome across the background genome of the C3H strain of the Recombinant Congenic Strain HcB-28/Dem. The HcB-28 strain was derived by crossings between mouse strains C3H/DISnA as background (displayed in grey) and C57BL/10 as donor (displayed in black). b) Mice (C3H/DISnA) were infected intranasally with *B. pertussis *strain B213. The lungs were removed seven days after inoculation, and the number of viable *B. pertussis *was determined. Bars represent the average number of bacteria in the lungs. Horizontal line indicates the significant difference between groups. Error bars indicate the Standard Deviation (SD).

We have used recombinant congenic mouse strains (RCS) as a tool to facilitate the mapping of low-penetrance quantitative trait loci that control complex traits such as a *B. pertussis *infection [7]. RCS are derived from two different inbred strains, the so-called background and donor strain. After two backcrosses and subsequent brother-sister mating, a set of RCS is created, with each strain containing 12.5% of the donor genome differently distributed across the background genome [8]. HcB/Dem RCS of mice are derived from two backcrosses of the inbred mouse strains C3H/DISnA (C3H) as background and C57BL/10ScSnA (B10) as donor strain, resulting in 12.5% B10 genome across the C3H genome. The genome of each HcB/Dem strain, thus, differs maximally 12.5% compared to the background strain (C3H) (Figure 1a) [9,10]. HcB-28 mice contained lower numbers of bacteria in their lungs seven days post-inoculation compared to C3H mice. Subsequent genotyping led to the identification of the three susceptibility loci, *Bps1, 2*, and *3*. Especially *Bps1 *displayed strong linkage with susceptibility to *B. pertussis *infection. The *Bps1 *locus is located on chromosome 12, spanning a region of 185 genes, of which one or more genes have a dominant positive effect on the clearance of *B. pertussis *in the lung, and/or the reduction of bacterial multiplication. However, the mechanism by which genes within this region influence the course of infection is not clear. Two other loci, *Bps-2 *and *Bps-3*, showed genetic interaction and are located on chromosomes 5 and 11, respectively [2].

Although *Bps-1 *has not yet been validated, we believe that the significance of this locus warrants further study. To further elucidate the role of host genetic factors, in particular the *Bps1 *locus, in *B. pertussis *infection, and to identify candidate genes within this region, we studied expression profiles in the lungs of mice following *B. pertussis *inoculation. The traditional approach for mapping genes in susceptibility loci is a combination of positional cloning and linkage analysis [11,12]. Although this strategy has proven to be effective [7,13], the approach is quite costly and animal-consuming. Previously we identified changes in gene expression in the lungs of C3H/DISnA mice after *B. pertussis *infection, and we especially focused on differentially expressed genes in the lungs of infected and non-infected mice located in *Bps1,-2*, and *-3 *[14]. We found that the expression of 1,841 genes was significantly changed upon *B. pertussis *infection. These genes are involved in various immune-related processes, such as the acute-phase response, antigen presentation, cytokine production, inflammation, and apoptosis. Nine of the differentially expressed genes are located in *Bps1*, 13 are located in *Bps-2*, and 62 are located in *Bps-3*.

In the present study we compared the gene expression profiles in the lungs of two mouse strains, i.e. HcB-28/Dem and C3H, which showed a different course of *B. pertussis *infection, in order to further identify candidate susceptibility genes without the need for positional cloning. We hypothesized that the phenotypic differences displayed by these mice in the response to *B. pertussis *can partly be explained by a different gene expression profile between the mouse strains, and that this approach could lead to the identification of candidate genes affecting the course of infection. Using this approach we reduced the number of candidate susceptibility genes within the *Bps1 *locus.

## Methods

### Experimental design

Forty-eight female HcB-28/Dem and 48 female C3H/DISnA (the background strain of the HcB-28/Dem) mice were intranasally inoculated with 2*10^7 ^colony forming units (CFU) of the streptomycin-resistant Tohama strain of *B. pertussis *(B213) in 40 μl Verwey medium (The Netherlands Vaccine Institute, Bilthoven, the Netherlands), or as a control with Verwey Medium only (total of 96 mice). One, three, and five days after inoculation, 8 infected and 8 control mice were euthanized. To remove blood from the lungs, mice were perfused with phosphate-buffered saline (PBS, Tritium Microbiology, Veldhoven, the Netherlands). Subsequently, the lungs and trachea were collected [15,16]. For RNA extraction, the right lung was collected in RNA stabilization reagent (RNAlater, Qiagen, Venlo, the Netherlands). For histological examination, the left lung was fixed intratracheally using 4% formalin.

The number of viable *B. pertussis *bacteria was determined in the trachea to confirm a proper infection [17] (Note that tracheal counts are not representative for bacterial clearance from the lungs.) To this end, approximately one centimeter of the trachea was collected in 500 μl Verwey medium. Bacterial suspensions were diluted in Verwey medium and the number of CFU was determined by plating on Bordet Gengou agar supplemented with 15% sheep blood and 30 μg/ml streptomycin (Tritium Microbiology). Plates were incubated for 4 days at 35°C and the resulting colonies were counted using a ProtoCOL Colony counter (Synbiosis, Cambridge, United Kingdom).

All *B. pertussis*-infected mice had between 10^2 ^and 10^5 ^CFU in the trachea during the first five days after inoculation, confirming an actual infection (data not shown).

For the analysis of intrinsic immunological and cellular differences between the two mouse strains, independent from infection, blood and spleen samples were collected from five untreated female mice of both strains.

### Animals

Breeding pairs of C3H/DISnA and HcB-28/Dem were supplied by the Department of Molecular and Cellular Biology, Roswell Park Cancer Institute, Buffalo, New York. Female mice were bred to the appropriate numbers at our animal testing facility in Bilthoven, the Netherlands. Mice were acclimatized for at least one week before the start of the experiments. Mice received standard laboratory food (SRM-A, Hope Farms, Woerden, the Netherlands) and tap water ad libitum. All animal experiments were approved by the Institute's Animal Ethics Committee and were performed according to NIH guidelines [18] and Dutch legislation.

### Clinical and pathological examinations

Mice were weighed before inoculation, and subsequently every day after inoculation to determine the relative change in weight. Lung weights were determined post mortem as quantitative parameter for lung inflammation. Formalin-fixed lungs were embedded in paraplast (Monoject Inc., St Louis, MO). Sections (5 μm) were stained with hematoxylin-eosin. Lung lesions were examined for infiltration of inflammatory cells in the peribronchiolar space (peribronchiolitis), infiltration of inflammatory cells in the alveoli (alveolitis), infiltration of inflammatory cells in the perivascular space (perivasculitis), hypertrophy of mucus-producing glands, free protein (exudate), and eosinophilia. Lung lesions were scored semi-quantitatively as absent, minimal, slight, moderate, marked, or strong, as previously described [19].

### Enumeration of cellular subsets

Spleens were harvested and weighed. Cell suspensions were prepared and the number of nucleated cells per spleen was determined using a Coulter Counter Z2 (Beckman Coulter, Mijdrecht, the Netherlands). The percentage of B- and T-cells, as well as T-cell subsets were determined using a fluorescence-activated cell sorter (FACS-Calibur, BD Biosciences, Alphen aan den Rijn, the Netherlands). T-cells were detected with phycoerythrin-labeled (PE) anti-CD3ε antibodies (Molecular probes, Invitrogen, Breda, the Netherlands). CD4+ T-cells were determined using allophycocyanin-labeled (APC) antibodies (Molecular probes) and CD8+T-cells were determined using fluorescein isothiocyanate-labeled (FITC) antibodies (Molecular probes). B-cells were determined using PE-labeled anti-CD19 antibodies (Molecular probes).

### Splenocyte proliferation test

The cell suspensions were used to measure the spontaneous (medium only) or stimulus-induced ^3^H-thymidine uptake. The cells were stimulated by adding 10 μg/ml Concavalin A (Con A, T-cell stimulus), 70 μg/ml lipopolysacharide (LPS, B-cell stimulus) or 15 μg/ml Lectin (B- and T-cell stimulus) to the culture medium. Cells were incubated for 72 hr at 37°C. ^3^H-thymidine was present during the final 24 hr of culture. See [20] for details.

### Hematology

The morphologic hemogram was determined using an ADVIA 120 Hematology system (Multispecies analyzer, Bayer, Mijdrecht, the Netherlands).

### Immunoglobulin quantification

Blood was collected in Vacuette Minicollect tubes (Greiner bio-one, Alphen a/d Rijn, the Netherlands), and serum was removed by centrifugation. Immunoglobulins were determined using the multiplex Beadlyte Mouse Immunoglobulin Isotyping Kit (Millipore, Billerica, MA) for the Luminex platform (Luminex, Oosterhoud, the Netherlands), as prescribed by the manufacturer.

### Transcription profiling

Microarray analysis experiments were performed as described previously [14]. Briefly, total RNA was extracted from lungs and amplified using the Amino Allyl MessageAmp II aRNA kit (Ambion Inc., Austin, TX). RNA amplification was performed to obtain more nucleic acid for labeling. This results in a stronger fluorescence signal and a better signal/noise ratio. Because of this, less experimental samples (and therefore animals) are needed to obtain sufficient statistical power. RNA samples from individual mice were labeled with Cy3. A common reference containing a RNA pool of all samples isolated was labeled with Cy5.

Microarray slides were spotted at the Microarray Department of the University of Amsterdam. The slides contain 21,997 65-mer oligonucleotides from the Sigma-Compugen Mouse oligonucleotide library, 192 additional 65-mer oligonucleotides of genes of which 185 are located in the *Bps1 *region spotted in duplicate (Isogen Life science, IJsselstein, the Netherlands), as well as appropriate controls and blank spots. The *Bps-1 *oligonucleotides were annotated according to NCBI mouse genome build 34.1; all other oligonucleotides were annotated according to the Sigma-Compugen Mouse oligonucleotide library.

At least 5 slides per group were used. Raw microarray signal data were normalized and analyzed in the R statistical software environment [21-23]. Significance of differences in gene expression between the experimental groups was calculated in R with ANOVA. The false discovery rate (FDR) was calculated according to Benjamini and Hochberg [24]. Gene expression data were visualized by hierarchical clustering (using Euclidian distance and Ward linkage) using GeneMaths (Applied Maths, St-Martens-Latem, Belgium). Gene categories were defined by Gene Ontology (GO) [25], Classification and enrichment according to GO categories or location were determined using DAVID/EASE [26,27], using EASE-scores as *P-*value for enrichment.

## Results

### Response of C3H and HcB mice to infection

We have previously examined clearance of *B. pertussis *from the lungs of infected RCS mice, and established that HcB-28 mice contained lower numbers of bacteria in their lungs seven days post inoculation compared to C3H mice (Figure 1b) [2]. To examine whether the genetic differences between "resistant" HcB-28 mice and "susceptible" C3H mice also result in a different transcriptional response to *B. pertussis *infection, we compared gene expression profiles of the lungs of infected and uninfected HcB-28 and C3H mice [2,14]. mRNA transcription profiles were determined in at least five animals per group.

**Figure 2:**
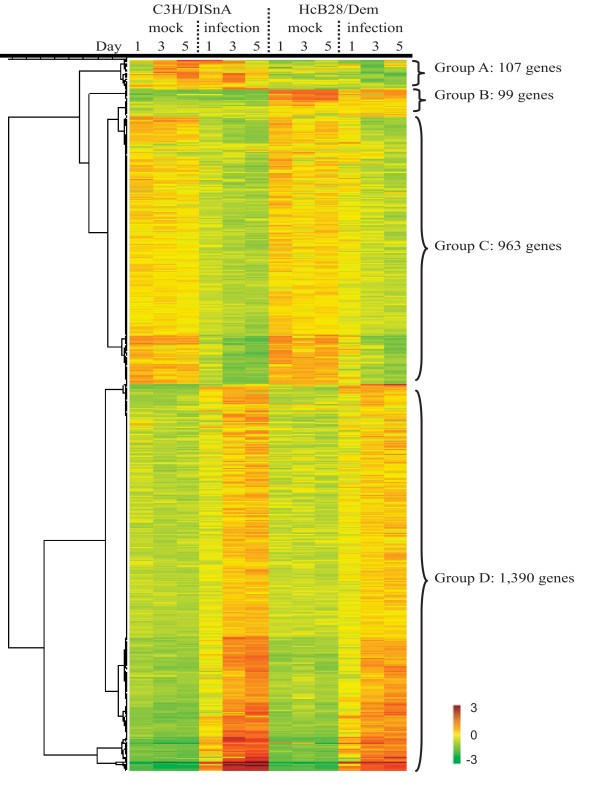
**Presentation of microarray data from mock-and *B. pertussis- *inoculated mice**. Analysis was performed on 2,559 genes whose expression was found to be significantly up- or down-regulated at an FDR < 0.05. Relatively increased gene-expression is presented in red while relatively decreased gene-expression is presented in green. The color scale shows log(2) ratios of gene-expression levels compared to the global average. ANOVA analysis identified 206 genes that were differentially expressed between the two mouse strains and 2,353 genes that were differentially expressed after infection. Hierarchical clustering of the 2,559 regulated genes results in four distinct groups, higher in C3H (A), higher in HcB-28 (B), down-regulated after infection in both strains (C) and up-regulated after infection in both strains (D).

The three mock-inoculated groups of each mouse strain, euthanized at days one, three and five, were found to be statistically indistinguishable from each other, i.e. both within the same strain and between strains (i.e. there were no genes with FDR < 0.6). Of the approximately 22k genes tested, 2,559 genes were significantly differentially expressed either in the two mouse strains or upon *B. pertussis*-inoculation (FDR <0.05, corresponding to P < 0.0058). The gene expression levels of all 2,559 genes are presented in additional file 1. The expression profiles of all genes are illustrated by hierarchical clustering in Figure 2. ANOVA analysis identified 206 genes (groups A and B) that were differentially expressed between the two mouse strains before inoculation, and 2,353 genes (groups C and D) that were differentially expressed in both strains after infection. Remarkably, there were no genes that were differentially expressed between the two mouse strains following inoculation. Thus, genes up- or down-regulated upon infection were identically regulated in both mouse strains despite their clear genetic differences and differences in *B. pertussis *susceptibility.

The 206 genes that were differentially expressed between the two strains were all unaffected by infection, but displayed a difference in basal expression level. Of the 206 strain-dependent genes, 107 genes were expressed at a higher level in C3H/DISnA mice (group A) and 99 genes were expressed at a higher level in the HcB-28/Dem mice (group B). Of the 2,353 infection-regulated genes, 963 were down-regulated upon infection (group C), and 1,390 were up-regulated upon infection (group D). All genes that were affected by *B. pertussis *infection followed a similar trend in time. One day post-inoculation differential gene expression was already observed, but the effect was stronger on days three and five post-inoculation. Thus there was no identifiable subset of genes that displayed a different kinetic trend in gene expression.

To assign functions to the differentially expressed genes, we classified them according to Gene Ontology (GO) categories. In addition, we determined enrichment for location and biological functions to identify pathophysiological processes involved in the response to *B. pertussis *inoculation. The most important GO categories, i.e. with the highest percentage differentially expressed genes, are summarized in Table 1. Many genes that were differentially regulated between the two strains of mice (groups A and B) are located on chromosome 12, predominantly in *Bps1 *(8 in group A and 9 in group B)[2]. All the 17 genes that were differentially expressed in the mouse strains C3H and HcB-28 and that are located in *Bps1 *are presented in Table 2, and may be considered as candidate susceptibility genes. Of these genes, the strongest differences in gene expression between the mouse strains were found for a cDNA sequence in group A (*BC022687*, up to 3.7-fold higher in C3H mice) and for Immunoglobulin heavy chain 1 in group B (*Igh-1*, up to 3.6-fold higher expression in HcB-28 mice). Eight genes within *Bps1 *(6 in group A and 2 in group B) showed a strong difference (i.e. at least two-fold) in gene expression between the two mouse strains, and map to the immunoglobulin heavy chain complex (*Igh*).

**Table 1:** Gene-ontology based classification of genes with different expression

	Group A	Group B	Genotype (A+B)	Group C	Group D	Infection (C+D)	Total on array
Acute-phase response	0 (0%)	0 (0%)	0 (0%)	0 (0%)	13 (59%)	13 (59%)	22
Antigen presentation	0 (0%)	0 (0%)	0 (0%)	0 (0%)	23 (72%)	23 (72%)	32
Apoptosis	0 (0%)	3 (1%)	3 (1%)	9 (3%)	50 (15%)	59 (18%)	324
Cell cycle	0 (0%)	1 (0%)	1 (0%)	30 (6%)	59 (12%)	89 (17%)	510
Chemokine Activity	0 (0%)	0 (0%)	0 (0%)	1 (3%)	24 (67%)	25 (69%)	36
*Mus musculus 12*^a^	12 (2%)	11 (2%)	23 (4%)	34 (6%)	38 (7%)	72 (13%)	560
*Bps-1 (Mus musculus 12)*	8 (4%)	9 (5%)	17 (9%)	6 (3%)	8 (4%)	14 (8%)	185
Complement activation	0 (0%)	1 (3%)	1 (3%)	1 (3%)	8 (26%)	9 (29%)	31
Cytokine Activity	0 (0%)	0 (0%)	0 (0%)	8 (4%)	56 (29%)	64 (33%)	192
Cytoskeleton	0 (0%)	2 (0%)	2 (0%)	48 (9%)	45 (8%)	93 (18%)	531
Development	5 (0%)	9 (1%)	14 (1%)	137 (10%)	118 (8%)	255 (18%)	1433
Immune response	1 (0%)	2 (0%)	3 (1%)	6 (1%)	176 (43%)	182 (45%)	408
Inflammatory response	0 (0%)	1 (1%)	1 (1%)	5 (4%)	52 (43%)	57 (47%)	121
Metabolism	3 (0%)	20 (0%)	23 (0%)	300 (6%)	405 (8%)	705 (15%)	4771
Muscle contraction	0 (0%)	0 (0%)	0 (0%)	14 (29%)	2 (4%)	16 (33%)	49

Listed in any of the above	19	29	48	453	656	1109	6574
Other	3	22	25	383	513	896	5639
Unannotated	85	48	133	127	221	348	9744
Total	107	99	206	963	1390	2353	21957

^a^Genes located on chromosome 12

**Table 2:** Candidate susceptibility genes

Symbol	Max.fold^a^	*P-*value	Group^b^	Description
2610204M08Rik	1.5	0.0000000	**A**	RIKEN cDNA 2610204M08 gene
BC022687	3.7	0.0005453		cDNA sequence BC022687
LOC544906^c^	2.6	0.0024627		similar to monoclonal antibody heavy chain
LOC382694^c^	2.8	0.0043821		similar to immunoglobulin heavy chain
LOC211331^c^	2.7	0.0002482		similar to Ig H-chain
LOC238440^c^	2.7	0.0016035		similar to IgE antibody heavy chain (VDJ)
LOC238448^c^	3.2	0.0000868		similar to Igh-VJ558 protein
LOC544911^c^	2.1	0.0000310		similar to heavy chain V region VH558 A1/A4 precursor

LOC432692	1.4	0.0005147	**B**	LOC432692
LOC544805^c^	3.5	0.0000050		similar to Ig heavy chain variable region precursor
1700001K19Rik	1.6	0.0002085		RIKEN cDNA 1700001K19 gene
Amn	1.7	0.0002930		amnionless
Ppp1r13b	2.2	0.0000019		protein phosphatase 1, regulatory (inhibitor) subunit 13B
Adssl1	1.5	0.0001518		adenylosuccinate synthetase like 1
Akt1	1.8	0.0000206		thymoma viral proto-oncogene 1
AI450948	1.5	0.0048316		expressed sequence AI450948
Igh-1a^c,d^	3.6	0.0000000		immunoglobulin heavy chain 1a (serum IgG2a)

^a^Maximum fold-change between genes that are located in *Bps1 *and that are significantly differentially expressed between C3H and HcB-28 mice.

^b^Hierarchical clustering as presented in figure 2, higher in C3H (A) or higher in HcB-28 (B).

^c^In the most recent annotation of the murine genome, eight probes map to the *Immunoglobulin heavy chain complex (Igh) *similar to Ig heavy chain variable region precursor.

^d^The oligo was designed based on accession number XM_484178 annotated as *Igh-1a*. Because this annotation is based on the reference C57BL/6 mice, the oligo can be considered as *Igh-1b *annotated.

Of the 2,353 genes that were differentially expressed after *B. pertussis *infection, 1,702 genes were identical to the genes we have described previously to be regulated upon *B. pertussis *infection [14]. Thus by examining additional mice, we identified approximately 650 additional genes regulated by *B. pertussis*, most of which were weakly up- or down-regulated. Most up-regulated genes upon *B. pertussis *infection (group D) are involved in immune- and inflammation-related processes or in generic processes, while most down-regulated genes (group C) are involved in non-immune processes such as muscle contraction.

### Hematologic and immunologic analyses in non-infected C3H and HcB-28 mice

To investigate possible differences in the basal immunological parameters between the two strains, we analyzed hematologic parameters, mitogen-induced splenocyte proliferation, and lymphocyte subset distributions. Because the two mouse strains displayed clear differences in basal gene expression, we wanted to examine if these differences led to alterations in blood cell composition or basal immune status. We observed a slightly higher number of platelets (*P *= 0.0003) in the C3H/DISnA mice (1.1*10^12^/l) compared to the HcB-28/Dem mice (9.6*10^11^/l), and a slightly higher number of white blood cells (*P *= 0.006) in the C3H/DISnA mice (7.3*10^9^/l) compared to the HcB-28/Dem mice (6.5*10^9^/l), but no differences in the numbers of red blood cells, reticulocytes, lymphocytes, neutrophils, eosinophils, and basophils (data not shown).

Splenocyte proliferation was determined by mitogen-induced ^3^H-thymidine uptake. There was no difference in proliferation between the two strains after *ex vivo *stimulation with Con A (T-cell stimulus), LPS (B-cell stimulus) or Lectin (B- and T-cell stimulus) (data not shown). We observed no differences in the number of CD19^+ ^(B), CD3ε^+ ^(T), CD4^+^(Th) and CD8^+ ^(CTL) cells (data not shown).

**Figure 3:**
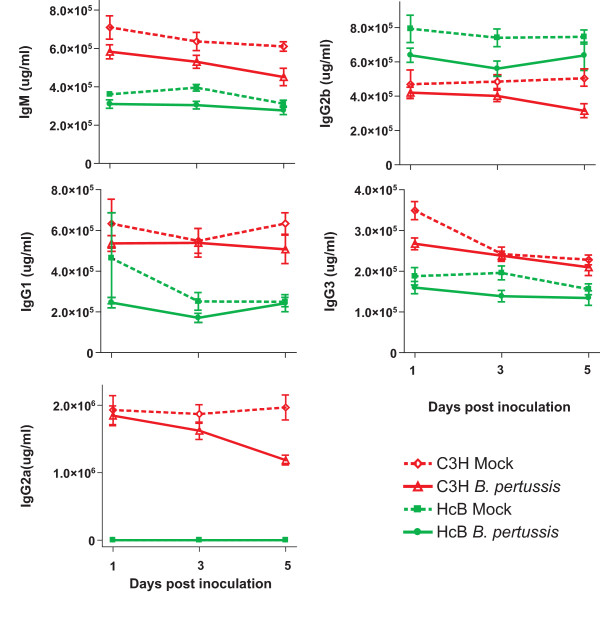
**Serum levels of immunoglobulin classes after *B. pertussis *infection**. Twenty-four mice per group were inoculated with either *B. pertussis *or with culture medium only (Mock), mice were subsequently euthanized at one, three or five days post inoculation. Blood was collected for the measurement of serum levels of immunoglobulin classes. C3H mice are presented in red and HcB mice are presented in green. Dashed lines represent mock-inoculated mice and continuous lines represent *B. pertussis*-inoculated mice. Error bars represent the standard deviation. No significant difference (*P *> 0.05, Bonferoni, ANOVA) was found between mock- or *B. pertussis*-inoculated mice of the same mouse strain, except for IgM at day 5 in C3H mice, IgG2a at day 5 in C3H mice, IgG2b at day 3 in HcB mice, IgG3 at day 1 in C3H mice and IgG3 at day 3 in HcB mice. All serum levels were significantly different (*P *< 0.05, Bonferoni, ANOVA) between the two strains of mice except for IgM at day 5, IgG1 at day 1 and IgG2b at all days for *B. pertussis *infected HcB mice compared to C3H mice.

Finally we determined levels of IgM and subclasses of IgG in the sera of the mock- and *B. pertussis*-inoculated mice (Figure 3). Infection did not affect Ig levels compared to mock-infected mice of the same strain. We did, however, observe a significant difference in the Ig subset distribution between the two mouse strains (either mock- or *B. pertussis*-inoculated). C3H mice had significantly higher serum levels of IgM, IgG1, IgG2a and IgG3, while HcB-28 mice had significantly higher IgG2b levels compared to C3H mice. Importantly, HcB-28 mice had no detectable levels of IgG2a in the serum at all, which is consistent with the difference in gene expression at the *Igh-1 *locus between the two mouse strains.

### Clinical and pathological findings in *B. pertussis*-infected C3H and HcB-28 mice

**Figure 4:**
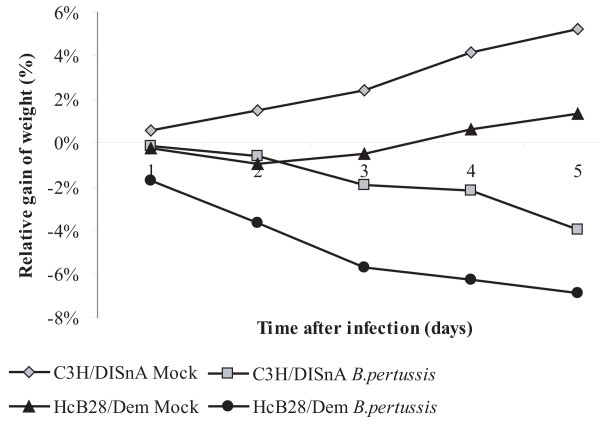
**Relative gain or loss of weight after inoculation**. Twenty-four mice per group were inoculated with either *B. pertussis *or culture medium only (Mock) and body weights were measured daily.

To examine whether the two mouse strains differed in weight loss after infection, we determined their body weights after infection. Infected mice lost three to four percent of their body weight during the first five days post-inoculation, while all mock-inoculated mice gained weight during these five days (Figure 4). No significant differences in the gain or loss of weight were observed between the two strains, irrespective whether they were infected or not.

As a quantitative endpoint for inflammation, we determined lung weights relative to body weight. All mock-inoculated mice had relative lung weights of approximately one percent five days after inoculation, while *B. pertussis-*infected mice had relative lung weights of up to three percent. This difference in relative lung weights due to *B. pertussis *infection is significant from day one till day five post-inoculation, but was similar in both mouse strains (data not shown).

**Figure 5:**
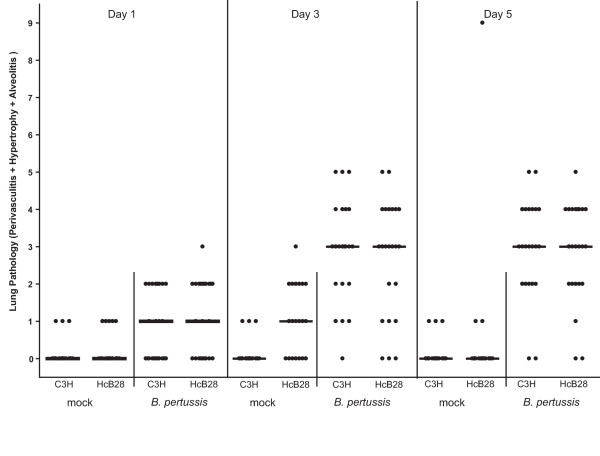
**Summary of lung pathology post mock- or *B. pertussis*-inoculation**. H&E stained slides were examined for alveolitis, perivasculitis and hypertrophy of mucus-producing glands. Lung lesions were scored semi-quantitatively as absent (0), minimal (1), slight (2), moderate (3), marked (4), or severe (5) per type of lesion and added up to calculate the pathology-score (range 0–15). Dots represent the pathology score per individual mouse, horizontal lines represent the groups median.

**Figure 6:**
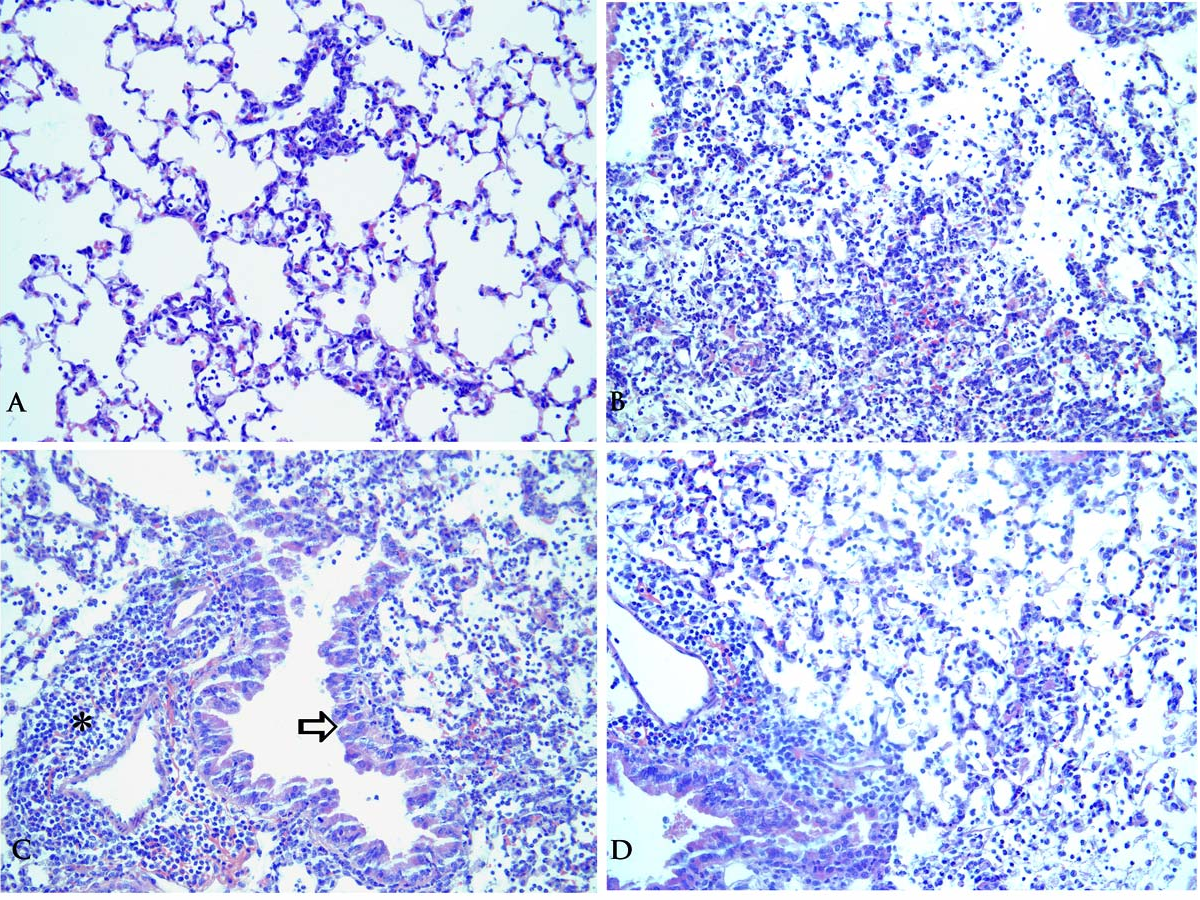
**Examples of lung pathology post *B. pertussis *inoculation**. Lung sections (H&E, obj x 20) from *B. pertussis *infected mice. **A: **HcB28 day 1, mild alveolitis (grade 2 on scale from 1–5), as seen by inflammatory cells in alveolar septa and spaces. **B: **C3H day 3, alveolitis and thickened septa (pneumonia) varying from grade 3 (upper, right) to 5 (bottom). **C: **C3H day 3, asterix: perivasculitis grade 5, and arrow: hypertrophy bronchiolar epithelium, grade 3. **D: **HcB28 day 3, no major differences in pathology compared to C3H (B and C).

Histopathological evaluation of lung sections was performed as a second parameter of lung inflammation. Intranasal inoculation of *B. pertussis *causes an acute inflammatory response that is characterized by influx of polymorphonuclear leukocytes (PMNs) and macrophages, starting in the perivascular and peribronchiolar areas on day one, and extending to alveolar walls and lumina on days three and five. No differences were observed in the histopathological lesions findings between the two mouse strains (Figure 5 and 6).

## Discussion

Studying genetic differences in susceptibility to *B. pertussis *infection may point to novel insights in the pathogenesis of this infection. We have previously identified *B. pertussis susceptibility locus-1 *(*Bps1*) in HcB-28 mice [2]. The *Bps1 *locus is located on chromosome 12, spanning a region of 185 genes and has a dominant positive effect on the clearance of *B. pertussis *from the lung. In this study we examined gene expression profiles in HcB-28 and C3H mice, which differ in their susceptibility to *B. pertussis*. Twelve and a half percent of the genomes of these mice are from a different genetic background [9,10]. The traditional approach for identification of relevant genes in susceptibility loci is a combination of positional cloning and linkage analysis [11,12]. This method has proven to be effective [7,13], but has disadvantages. We have therefore chosen for an alternative strategy. Using this approach we attempted to identify candidate susceptibility genes that control the difference between these two mouse strains. We hypothesized that the difference in susceptibility to *B. pertussis *infection could (partly) be explained by a different gene expression profile between the mouse strains.

We have previously shown that *B. pertussis *infection in C3H mice induces a wide transcriptional response, which appears to be partly specific for *B. pertussis *and partly non-specific [14]. This study revealed that 1,841 genes are differentially expressed in the lungs of mice after *B. pertussis *inoculation, and most up-regulated genes are involved in immune- and inflammation-related processes or in generic processes, while most down-regulated genes are involved in non-immune processes. In the present study, we found that HcB-28 and C3H mice showed a similar gene expression profile upon infection and identified approximately 650 additional genes regulated by *B. pertussis*, most of which were weakly up- or down-regulated. However, the gene expression profiles and enrichment for GO categories were identical as described in the previous study. A substantial number of genes and pathways suggest a central role of PMN recruitment and activation in the pathogenesis of *B. pertussis *infection. The transcriptional profiles further indicate in particular the significance of TLR activation and apoptosis [14]. The reason for the detection of the 650 additional genes, besides the strain differences, is that by adding an extra mouse strain the number of samples doubled increasing the power of detection. Hundred and thirty-nine genes which we have described previously to be regulated upon *B. pertussis *infection were not detected in the present study. These genes were borderline significantly regulated (median FDR of 0.03) and only slightly induced (median 1.4-fold). These genes are therefore probably less important in the host response to *B. pertussis *infection. The finding that this list of 139 genes does not show significant enrichment for any GO-term, including immunological terms, corroborates this.

Although the two mouse strains differ in 12.5% of their genome (12.5% of the genomes of these mice are from a different genetic background), we observed no marked differences in their phenotypical characteristics other than the previously observed difference in bacterial numbers in the lungs after infection (Figure 1b). C3H mice did have slightly higher numbers of circulating platelets and white blood cells compared to the HcB mice, but the cellular proportions, as well as the proliferation of splenocytes was identical for both strains. There was also no significant difference observed in body weight, lung weight and histopathological findings in response to *B. pertussis *infection between the two mouse strains. The major difference is that HcB-28 mice did not have detectable IgG2a serum levels.

We observed 206 genes that were differentially expressed between the two mouse strains, but these genes were identically expressed in mock- or *B. pertussis*-inoculated mice. The majority of these genes (65%) are unannotated. These unannotated genes tend to have no GO functional annotation, because they are not "regular" protein-coding genes and many of them are not (sufficiently) mapped to a chromosomal locus to warrant including them under chromosome 12 or *Bps-1*. The mentioned 23 genes, which were mapped to chromosome 12, are significant at *P *= 5.29e-014 (Fisher exact probability, Bonferroni correction for multiple testing). The same test applied to all other mouse chromosomes yielded *P *values > 0.05. Twenty-three out of the 206 genes were located on chromosome 12, which can be explained by the fact that approximately one fifth of the genetic variation between the two mouse strains is due to variation on chromosome 12 [2]. Because the genes that were differentially regulated between the two mouse strains only showed differences in expression before infection, it appears likely that such intrinsic differences in gene regulation are involved in determining differences in susceptibility to *B. pertussis *infection. Alternatively, such genetic differences may be explained by genes that are not differentially regulated between these two strains of mice, or by processes at present not fully characterized and possibly involving differential expression of genes by mechanisms such as microRNA's. Remarkably, 17 of these genes were located in the *Bps1 *region, 8 of which mapped to the *Igh *complex. Among these 8 genes were the *Igh-1 *gene and genes that encode for Ig heavy chain variable regions. We observed a significantly higher expression (up to 2.8-fold) of the gene variant of *Igh-1 *isotype b in HcB mice compared to C3H mice. The Igh locus is genetically polymorphic and very complex [28]. The *Igh-1 *gene exists in 2 major genetic variants (*Igh-1a *and *Igh-1b*) with 83.8% similarity [29]. The *Igh-1a *allele codes for the heavy chain of IgG2a while the *Igh-1b *allele codes for the heavy chain of IgG2c [30]. Mouse strains such as C57BL/6 and C57BL/10 (the donor strain of the HcB mice) only contain the gene variant *Igh-1b *and are therefore incapable of producing IgG2a, while mouse strains such as BALB/c only contain the gene variant of *Igh-1a *and are therefore incapable of producing IgG2c [29,30]. The oligo for *Igh-1 *spotted on the microarray, was designed based on accession number XM_484178 annotated as *Igh-1a*. Because this annotation is based on the reference C57BL/6 mice, this oligo can be considered as *Igh-1b *annotated. In the sera of HcB mice we detected no IgG2a while C3H mice had significant titers of IgG2a, thereby confirming the expression results. It is tempting to speculate if and how genes within the *Igh *complex may affect differences early in the course of *B. pertussis *infection. Possible mechanisms may include differences in transcriptional gene regulation affecting immune responsiveness, different function of the IgG2a or c isotypes, or different usage of V chains. This latter possibility might imply the existence of "natural antibodies" reacting with *B. pertussis *epitopes. It has previously been shown that genes within the *Igh-1 *locus are predominantly associated with the course of a herpes simplex virus type-1 (HSV-1) infection in mice by an unknown mechanism [31-33]. Pro-inflammatory cytokines such as IL-1β, IL-4, IL-6 and IL-7 participate in this infection [34]. Interestingly, natural killer cell activity appears to be regulated by the *Igh-1 *locus but could not simply explain the differences in HSV-1 susceptibility [33]. *Igh*-linked genes have further been implicated in T suppressor cell activity [35,36].

## Conclusion

In this study we reduced the number of candidate susceptibility genes within the *Bps1 *locus by microarray analysis. Gene expression changes upon *B. pertussis *infection appear highly identical between C3H and HcB-28 mouse strains despite the different course of *B. pertussis *infection in these strains. Because the genes that were differentially regulated between the mouse strains only showed differences in expression before infection, it appears likely that such intrinsic differences in gene regulation are involved in determining differences in susceptibility to *B. pertussis *infection. Alternatively, such genetic differences in susceptibility may be explained by genes that are not differentially regulated between these two mouse strains or by processes other than differential gene expression. Genes in the *Igh *complex, among which *Igh-1*, may be likely candidates to explain differences in susceptibility to *B. pertussis*. Further work should establish the role of the *Igh *complex in *B. pertussis *infection and determine its mode of action.

### Supplementary data

Raw data as well as the detailed description of the experiment was uploaded to the freely accessible online database ArrayExpress [37]. Expression levels of all 2,559 genes are presented in the additional files.

## Competing interests

The author(s) declares that there are no competing interests.

## Authors' contributions

SB: carried out the infection and microarray studies and wrote the manuscript. RJV: participated in the study design and coordination and helped to draft the manuscript. JLAP: participated in the design of the microarray analysis and performed the statistical analysis. ERG: carried out the immunoassays. PWW: evaluated the lung pathology. TMB: participated in the design of the microarray analysis. PD: responsible for the genetic model of recombinant congenic mice. HJK, FRM, and BH: participated in the study design and coordination. TGK: conceived the study, and participated in its design and coordination and helped to draft the manuscript. All authors read and approved the final manuscript.

## Supplementary Material

Additional file 1 Comparative gene expression profiling in two congenic strains of mice following Bordetella pertussis infection. The data present the expression levels of all 2,559 genes. Click here for file
